# Communication Priorities and Experiences of Caregivers of Children With Cancer in Guatemala

**DOI:** 10.1200/GO.21.00232

**Published:** 2021-11-08

**Authors:** Dylan E. Graetz, Silvia Elena Rivas, Huiqi Wang, Yuvanesh Vedaraju, Ana Lucia Fuentes, Annie Caceres-Serrano, Federico Antillon-Klussmann, Meenakshi Devidas, Monika L. Metzger, Carlos Rodriguez-Galindo, Jennifer W. Mack

**Affiliations:** ^1^ St Jude Children's Research Hospital, Memphis, TN; ^2^ Unidad Nacional de Oncología Pediátrica, Guatemala City, Guatemala; ^3^ Francisco Marroquin University School of Medicine, Guatemala City, Guatemala; ^4^ Dana Farber Cancer Institute/Boston Children's Hospital, Boston, MA

## Abstract

**PURPOSE:**

Although > 90% of children with cancer live in low- and middle-income countries, little is known about communication priorities and experiences of families in these settings. We examined communication priorities and the quality of information exchange for Guatemalan caregivers of children with cancer during diagnostic communication.

**METHODS:**

A cross-sectional survey including items used in pediatric communication studies from high-income countries and novel questions was verbally administered to 100 caregivers of children with cancer in Guatemala.

**RESULTS:**

Guatemalan caregivers prioritized communication functions of exchanging information (99%), fostering healing relationships (98%), decision making (97%), enabling self-management (96%), and managing uncertainty (94%) over responding to emotions (66%) and cultural awareness (48%). Almost all caregivers wanted as many details as possible about their child's diagnosis and treatment (96%), likelihood of cure (99%), and late effects (97%). Only 67% were always given the information they needed without asking for it, and most caregivers sometimes (56%) or always (18%) had questions they wanted to discuss but did not. Approximately half of the caregivers (54%) correctly identified their child's diagnosis, primary site, disease extent (localized *v* metastatic), proposed treatment length, and treatment intent (curative *v* palliative). Caregivers of children with leukemia were more likely to correctly identify all attributes than those whose children had solid tumors (*P* < .001).

**CONCLUSION:**

Caregivers in Guatemala prioritize many of the same aspects of diagnostic communication as parents in the United States, and experience similar challenges. Shared communication values offer potential for adaptation of communication interventions across settings with varying resources and diverse cultures.

## INTRODUCTION

In high-income countries, high-quality pediatric cancer communication improves trust, facilitates decision making, and decreases patient and caregiver distress.^1-3^ Less is known about patient- and family-centered communication in low- and middle-income countries (LMICs), where approximately 90% of children with cancer live.^4^ Most pediatric cancer communication studies have been conducted in the United States and Western Europe.^5^ In LMICs, suboptimal communication contributes to delayed diagnosis and treatment abandonment,^6^ a leading cause of treatment failure for children with cancer.^7,8^ Thus, in addition to improving the quality of care, a deeper understanding of communication processes has the potential to improve outcomes in LMICs for children with cancer and their families.

**CONTEXT**

**Key Objective**
What do Guatemalan caregivers of children with cancer prioritize during diagnostic communication, and how are these priorities met?
**Knowledge Generated**
Caregivers in Guatemala prioritized many of the same elements of communication as those in the United States, and almost all caregivers wanted as many details as possible regarding their child's diagnosis and treatment, likelihood of cure, and late effects. Nevertheless, many caregivers were not given the information they needed without asking for it, many held back questions, and ultimately only about half correctly understood all aspects of their child's diagnosis and treatment plan.
**Relevance**
Shared communication values and challenges across diverse communities suggest communication interventions could be adapted and used across a range of resource settings.

We sought to examine pediatric cancer communication in Guatemala, a middle-income country with a single large pediatric cancer center, Unidad Nacional Oncología Pediátrica (UNOP). Guatemala was chosen for this study in part because of its cultural diversity; 24 distinct ethnic groups comprise 40% of the population.^9^ In addition, UNOP uses an interdisciplinary team of psychosocial providers from the time of diagnosis to assess and mitigate the risk of treatment abandonment.^10^ The overarching goal of this study was to understand caregiver priorities for communication about childhood cancer as well as their experiences with communication, including met and unmet needs. We drew on the United States National Cancer Institute's (NCI's) proposed six key functions of patient-centered communication in cancer care: exchanging information, fostering healing relationships, making decisions, managing uncertainty, enabling patient self-management, and responding to emotions.^11^ Although these functions were developed for medical oncology, all six have been applied to pediatric oncology in high-resource settings.^12^ Nevertheless, the extent to which these priorities are held by families in LMICs has not been evaluated, and we know little about the effectiveness of current communication processes. This study aimed to assess communication priorities and experiences of caregivers of children with cancer in Guatemala, as well as assessing the effectiveness of information exchange, including whether caregivers were able to correctly report their child's diagnosis, tumor location, extent of metastatic disease, treatment plan, and treatment intent.

## METHODS

### Setting and Participants

UNOP, Guatemala's national pediatric cancer center, is in Guatemala City. Approximately 500 cases of childhood cancer are diagnosed annually at UNOP, where overall survival is 67%.

Eligible participants included Spanish-speaking caregivers (defined as a parent or primary guardian) of pediatric patients (age ≤ 18 years) diagnosed with any type of cancer in the 8 weeks before survey administration. We targeted a sample size of 100 based on an a priori power calculation demonstrating 80% power (α = 5%) to detect an effect size of 0.1. One caregiver per child was offered participation in the study. Of 104 caregivers approached, 100 (96%) agreed to participate. Written informed consent was obtained for all participants. This study was performed in compliance with international regulations for protection of human research subjects and approved by oversight boards at St Jude and UNOP.

### Survey Development and Data Collection

A cross-sectional survey was developed using items established for pediatric communication research in high-income countries,^3,13-15^ and novel questions created by members of the research team with years of clinical experience engaging with the study population. The survey was developed in English, translated into Spanish, and reviewed by bilingual members of the research team. The survey was verbally administered face-to-face in Spanish by members of the research team (A.L.F and A.C.-S.) and psychologists not involved in the care of participant children (see Acknowledgment). The Spanish survey was pilot-tested with 23 caregivers, including a subset with very low literacy. Face and construct validity were assessed during piloting through cognitive interviewing including follow-up probes and inquiry regarding comprehension of specific terms. Survey items were iteratively revised based on piloting to improve clarity until interviews demonstrated success without further need for modification. The survey was backtranslated into English by bilingual members of the study team to ensure the original intent of questions was preserved.

**FIG 1:**
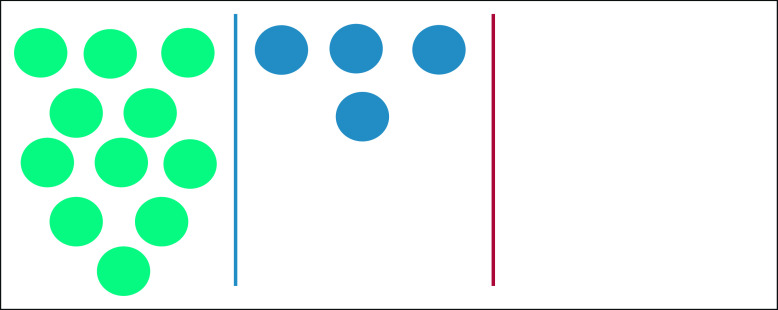
Visual Likert scale aid. A visual aid developed during pilot testing and used during survey administration in correlation with 3-item Likert scale response options. The teal circles on the left correspond with response options of very or always, the blue circles in the middle correspond with response options of somewhat or sometimes, and the blank area on the right following the red line corresponds with not at all or never.

*Communication priorities* were assessed using existing items^13^ structured around the six NCI communication functions. Caregivers were asked “How important is it to you that doctors and other health professionals explain things in a way you can understand?” (exchanging information), “are open and honest with you?” (fostering healing relationships), “involve you in making decisions about your child's care?” (making decisions), “help you deal with the uncertainties related to your child's cancer?” (managing uncertainty), “help you understand ways to take care of your child while dealing with cancer?” (enabling self-management), and “pay attention to your emotions and feelings?” (responding to emotions). An additional item was developed to address cultural awareness: “How important is it to you that doctors and other health professionals ask about your culture, background, and beliefs?” Original questions included a 5-point Likert scale. During pilot-testing, some caregivers were unable to distinguish between the five response options, whereas others consistently picked extreme answers, reducing variability. We collapsed response options to a 3-point Likert scale of very, somewhat, and not at all. Pilot-testing using the 3-point scale revealed ongoing difficulty for some parents. We added a visual aid that was successful in facilitating understanding and consistent ability to respond across literacy levels (Fig 1).

Priorities around information exchange were evaluated using questions developed in high-income countries.^14^ Caregivers were asked “What is your preference for details of information about your child's diagnosis and treatment?” with response options, “I prefer not to hear a lot of details,” “I want to hear details only in certain situations; in other situations I do not want to hear the details,” and “I want to hear as many details as possible in all situations relating to my child's cancer and its treatment.” Caregivers were asked “How important is it to you to know about your child's likelihood of being cured?” and “about the likelihood that cancer or its treatment may affect your child's life in the future?” Response options, adapted through pilot-testing, included “I prefer not to know,” “It doesn't matter to me if I know,” or “It is important for me to know.”

*Communication experiences* were assessed using questions corresponding to those about priorities.^13^ Caregivers were asked “How much do doctors and other health professionals make you feel comfortable asking questions?,” “have open and honest communication with you?,” “give you information and resources to help you make decisions?,” “help you deal with the uncertainties about your child's cancer?,” “make sure you understand the steps in your child's care?,” and “talk with you about how to cope with any fears, stress, and other feelings?.” A novel item was added regarding cultural awareness: “How much do doctors and other health professionals consider your culture, background, or religious beliefs when planning treatment for your child?” Adapted response options included always, sometimes, and never, using the visual aid used for priority-related questions.

Additional questions included, “Overall, how satisfied are you with the communication with your doctors and other health professionals?” (response options: not at all satisfied, fairly satisfied, and very satisfied), “How often do you feel like you are given the information that is important to you without needing to ask for it?,” and “When you see your child's doctor, how often do you have questions about your child's care that you want to discuss but do not?” (response options: always, sometimes, and never).

*Objective measures of caregiver understanding* focused on attributes of diagnosis and treatment planning. Open-ended questions included: “What is the name of your child's illness?,” “Where in your child's body is the [*use word caregiver used to describe illness*] located?,” and “Has the [*caregiver's word*] spread to other places in the body?” Multiple-choice questions asked caregivers “How long do you expect your child's treatment to last?” (response options: less than 6 months, 6 months to 1 year, more than 1 year but less than 2 years, or 2 years or more) and “Which of the following will be involved in the treatment of your child's cancer?” (response options: chemotherapy, surgery, and/or radiation treatment; caregivers choose all that applied). Finally, caregivers were asked about their medical team's main treatment goal and picked one response option: “to cure my child's cancer,” “to help my child live longer,” and “to decrease symptoms from the cancer.”

*Sociodemographic information* included survey questions on participants' sex, relationship to the child, languages spoken, ethnicity, religion, and belief in Mayan spirituality (including the Mayan value system and cosmologic beliefs). Additional sociodemographic information including parents' education, monthly household income, and travel distance to the cancer center was obtained from the social work intake form.

*Medical information* obtained from record review included the child's diagnosis, primary tumor location, metastatic sites, treatment plan (including modalities and duration of treatment), and treatment intent (documented as curative or palliative), all of which are standard aspects of diagnostic documentation at UNOP.

### Data Analysis

Sociodemographic information, communication priorities and experiences, and objective measures of information exchange were analyzed descriptively. Chi-square or Fisher's exact test was used to compare proportions between groups. McNemar's test was used to assess marginal asymmetry between priorities and experiences.

Responses regarding diagnosis, tumor location, metastatic disease, treatment intent, and duration were compared with information extracted from medical records to assess caregiver knowledge of disease and treatment. For diagnosis, tumor location, metastatic status, and treatment duration, a caregiver response matching the medical record was considered accurate. For treatment intent, caregiver responses of life extension or symptom reduction were considered palliative.

A summary variable was created using the number of accurate caregiver responses about diagnosis, tumor location, metastatic disease, length of treatment, and treatment intent with one point for each accurate response, for a total score of 0-5. For caregivers of patients with leukemia, this score ranged from 0 to 4 as answers regarding metastatic disease were excluded. The summary variable was dichotomized into caregivers who gave all (5/5; 4/4) accurate responses, versus those who had ≥ 1 inaccurate response. Univariate logistic regression was used to assess the impact of sociodemographic characteristics on the dichotomized summary variable.

## RESULTS

This study included 100 caregivers of children with cancer. Most respondents were parents (76% mothers and 22% fathers). A quarter of participants (25%) identified as Indigenous, and an additional 19% identified as mixed race. The remaining participants (56%) self-identified as *ladino*, indicating no identification with indigenous populations in Guatemala. Complete demographic information for caregivers and children is shown in Table 1.

**TABLE 1:**
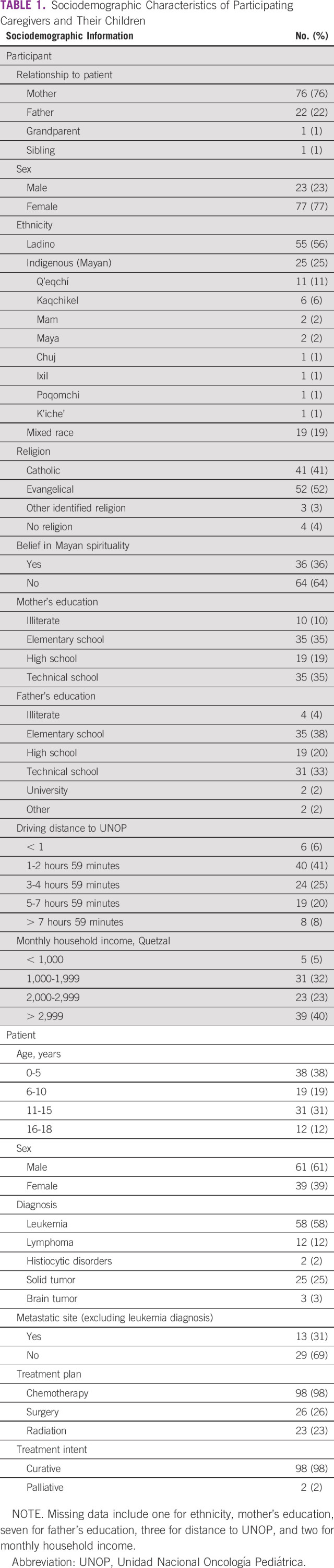
Sociodemographic Characteristics of Participating Caregivers and Their Children

Most caregivers considered it very important that their medical team explained things in a way they could understand (99%), was open and honest with them (98%), involved them in making decisions about their child's care (97%), helped them understand ways to take care of their child while dealing with cancer (96%), and helped them deal with uncertainties related to their child's cancer (94%). Fewer caregivers thought it was very important that their medical team pay attention to their emotions and feelings (66%; *P* < .001) or ask about their culture, background, or beliefs (48%; *P* < .001).

**FIG 2:**
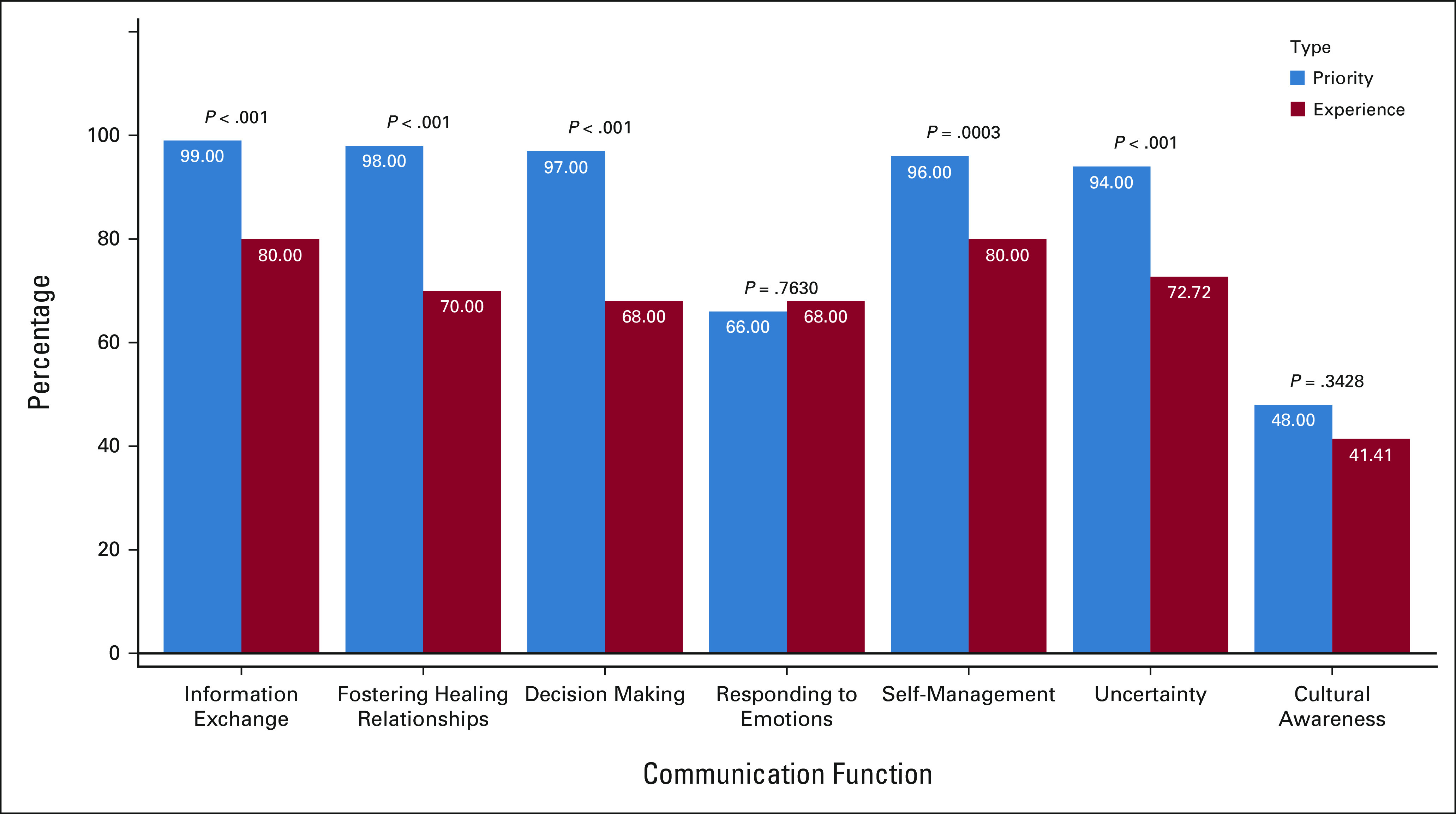
Priorities and experiences. Histogram demonstrating the percentage of parents who prioritized and experienced each communication function. McNemar's test was used to assess marginal asymmetry between priorities and experiences.

Overall, 83% of caregivers were very satisfied with the communication of their medical team. However, caregivers' communication experiences did not always match their priorities. For example, whereas 99% of caregivers prioritized information exchange, only 80% reported that they always felt comfortable asking questions (*P* < .001, Fig 2). Although 98% valued openness and honestly, only 70% always experienced open and honest communication (*P* < .001). Priorities similarly exceeded experiences for making decisions (97% *v* 68%, *P* < .001), enabling self-management (96% *v* 80%, *P* < .001), and managing uncertainty (94% *v* 73%, *P* < .001).

Almost all Guatemalan caregivers (96%) wanted as many details as possible in all situations relating to their child's cancer and treatment. Nearly all considered it important to know the likelihood of cure (99%) as well as the likelihood that their child's cancer or its treatment would affect the child's future (97%). However, only 67% of caregivers were always given information that was important to them without asking for it, and most caregivers sometimes (56%) or always (18%) had questions they wanted to discuss with their child's doctor but did not.

Compared to medical records, most caregivers correctly identified their child's diagnosis (92%) and tumor location (97%). Of caregivers with children diagnosed with cancers other than leukemia (N = 42), 81% understood whether their child's disease was metastatic. All caregivers whose children required chemotherapy correctly understood this; fewer correctly identified surgery (74%) or radiation (36%) as part of their child's planned therapy. Most caregivers (71%) identified an approximate length of treatment planned for their child's cancer consistent with the records, and 89% appropriately identified treatment intent (Table 2). Of 11 caregivers who misunderstood intent, 10 thought the team's primary goal was palliative when documentation described curative intent. About half of the caregivers (54%) had a view of all aspects of their child's cancer that was consistent with what was documented in the child's medical record.

**TABLE 2:**
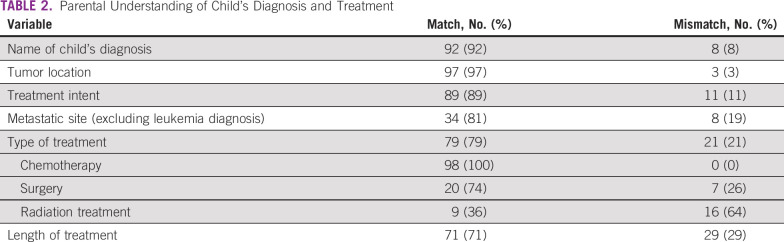
Parental Understanding of Child's Diagnosis and Treatment

In univariate analyses, understanding all aspects of diagnosis and treatment was not associated with caregiver sex, ethnicity, level of education, or family income. However, caregiver understanding was associated with having a child diagnosed with leukemia relative to a solid tumor (odds ratio, 10.5; 95% CI, 3.37 to 32.72; *P* < .001; Table 3).

**TABLE 3:**
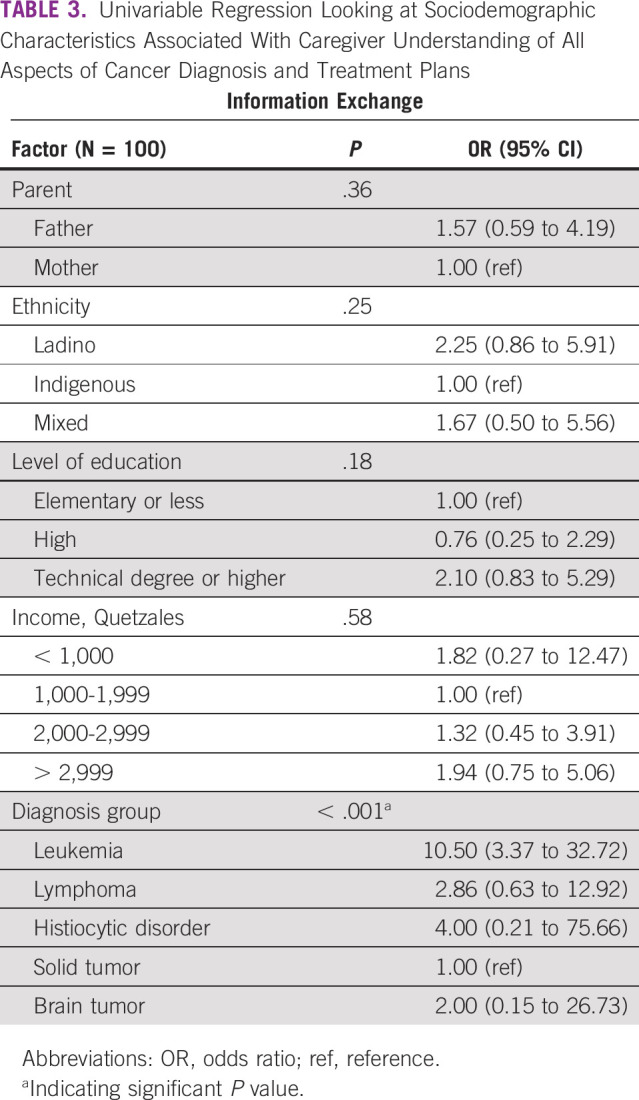
Univariable Regression Looking at Sociodemographic Characteristics Associated With Caregiver Understanding of All Aspects of Cancer Diagnosis and Treatment Plans

## DISCUSSION

Although children in LMICs comprise 90% of childhood cancer cases, we know little about the communication needs and experiences of their caregivers. In addition to a potential role in treatment acceptance and adherence, patient- and family-centered communication is a core aspect of high-quality care; communication allows caregivers to be informed about the child's condition, to fully participate in decisions about care, and to be supported through the challenges of diagnosis and treatment. Understanding communication experiences is thus an important underpinning of efforts to improve childhood cancer care around the world.

Since communication values may be culturally determined,^16^ we began our approach by investigating communication in a single country with a diverse population. We found that many patient-centered communication functions defined by the NCI were relevant to caregivers in Guatemala, suggesting that this model has utility for pediatric cancer patients and families in different socioeconomic and cultural environments, and that families in the very different settings of Guatemala and the United States may share a common set of priorities.

Almost all Guatemalan caregivers identified information exchange as an important aspect of diagnostic communication. Like parents in the United States,^14^ Guatemalan caregivers wanted as much details as possible regarding their child's diagnosis, treatment, potential for cure, and late effects. However, about one third of caregivers did not receive the information they considered important without asking for it, and most caregivers held back questions. In addition, only about half of the caregivers correctly understood basic aspects of their child's diagnosis and treatment, suggesting caregiver perceptions of insufficient information exchange are real.

Although the desire for information may be similar between caregivers in Guatemala and the United States, cultural factors affect the processes for information exchange. These challenges offer opportunity to develop systems that ensure caregivers have the knowledge they need. Caregivers in all settings may not know the questions to ask during initial visits with medical teams, or may be intimidated by hierarchical medical environments, which are more pronounced in LMICs.^17^ In Guatemala, cultural norms make it particularly difficult for female caregivers to ask questions.^18,19^ Additionally, time constraints are exacerbated by limited resources, and caregivers may not be given sufficient space to reflect or ask questions.

UNOP serves many families with low literacy,^20^ and specifically low health literacy, which may contribute to misunderstandings. Many caregivers in Guatemala are bilingual; however, their primary language is not Spanish. Most diagnostic communication at UNOP occurs in Spanish, with limited resources for interpreters, creating additional communication challenges. Furthermore, factors that limit caregiver questions, including time constraints and provider-patient hierarchy, likely contribute to caregiver misunderstanding. Notably, we were unable to identify major sociodemographic factors associated with caregiver understanding. Education, for example, was not a determinant of understanding, suggesting that challenges are not simply a result of low literacy.

Caregivers of children with solid tumors were less likely to fully understand their child's diagnosis and treatment plan than children with leukemia, and there are many potential factors contributing to this finding. We only assessed understanding of metastatic disease among caregivers of children with solid tumors, given the challenges of making this determination in leukemia. In addition, children with solid tumors are more likely to receive multimodal treatment rather than chemotherapy alone, which could in part explain our findings. Uncertainties surrounding diagnosis and treatment of solid tumors may further complicate information exchange, and the concept of appearance-altering surgeries is devastating for many Guatemalan families,^21^ which may influence their acceptance of information surrounding its necessity. Finally, leukemia is more likely to be discussed among families at UNOP, given its relative prevalence compared with solid tumors. Further research is needed to explore potential barriers to information exchange specific to the solid tumor population.

This study has several potential limitations. It was conducted within 8 weeks of diagnosis and thus, does not speak to communication priorities or experiences throughout the rest of the cancer care continuum. Only one caregiver was surveyed for each child with fewer fathers represented than mothers, and pediatric patients were not included in this study. Priorities for communication and information exchange may vary between family members, and our study fails to capture these complex dynamics. In addition, caregivers of patients with hematologic malignancies make up > 50% of participants; caregivers of children with solid or CNS tumors may be under-represented. However, our sample is reflective of the proportions expected at UNOP, where 63% of new cancer diagnoses are hematologic. Our survey was administered only in Spanish. Although most children in Guatemala have at least one caregiver who speaks Spanish, this may have created selection bias. Regression analysis was limited by sample size, particularly in certain subgroups. Finally, this study was conducted at one center in Guatemala, an upper middle–income country and was limited to the population of patients who presented to this center for care. Many of our findings are consistent with work in high-income countries, suggesting that there may be universally prioritized aspects of communication; however, further studies are necessary to determine which of our findings are generalizable to other populations within Guatemala and other LMICs, and which are specific to the study population.

In conclusion, findings from this study demonstrate that the NCI-defined functions and framework for communication are applicable to caregivers in Guatemala and suggest that there may be universally shared communication values. Further work is necessary in settings that are culturally and geographically diverse from Guatemala and the United States to explore this possibility. This work also demonstrates that Guatemalan caregivers experience some of the same challenges to information exchange experienced by parents in high-resource settings, encouraging future research to explore how interventions to improve communication may be adapted from one cultural context to another.
